# The effect of a family-centered empowerment model with traditional Chinese medicine health education on self-management in community-dwelling older adult hypertensive patients with a chance health locus of control

**DOI:** 10.3389/fpubh.2026.1743254

**Published:** 2026-07-30

**Authors:** Fang Li, Mengwei Li, Shaofan Guo, Yuzhong Cao, Qingjin Zhang, Tengteng Cheng, Yingying Li

**Affiliations:** 1 College of Nursing, Gansu University of Chinese Medicine, Lanzhou, Gansu, China; 2 Department of Endocrinology, Shenzhen Hospital of Integrated Traditional Chinese and Western Medicine, Shenzhen, Guangdong, China; 3 Gansu Provincial Maternity and Child-Care Hospital/Gansu Provincial Central Hospital, Lanzhou, Gansu, China; 4 Department of Critical Care Medicine, Affiliated Hospital of Gansu University of Chinese Medicine, Lanzhou, Gansu, China

**Keywords:** aged, family-centered empowerment, health education, HTN, patient education, self-management

## Abstract

**Background:**

Despite much evidence showing the effectiveness of structured self-management education for patients with hypertension (HTN), poor blood pressure control remains a challenge among chance health locus of control (CHLC) older adults with HTN.

**Objective:**

We explored the effect of a health education intervention based on the Family-Centered Empowerment Model (FCEM) on the self-management of opportunistic older adults with HTN in Lanzhou, China.

**Methods:**

We conducted a 2-armed, randomized controlled trial using a multistage sampling method in China, Lanzhou, from May 2023 to January 2024. A total of 60 patients were randomly assigned to receive either community nurse–led HTN education based on the FCEM or general community HTN education. Blood pressure, Body Mass Index (BMI), and multidimensional health locus of control (MHLC) Scale Scores, and HTN self-management scores were compared at baseline and at 3 and 6 months after the intervention.

**Results:**

Analyses were conducted on the 58 patients completing follow-up. In the experimental group, the systolic blood pressure (SBP) (*t* = −3.325, *P* = 0.002), diastolic blood pressure (DBP) (*t* = −5.110, *P* < 0.001), and the Chance control dimension of MHLC Scale scores (*t* = −4.781, *P* < 0.05) decreased; the scores on internal control dimensions (*t* = 8.493, *P* < 0.001) and self-management scores (*t* = 4.406, *P* < 0.001)improved compared with the control group at 6 months postintervention. There was a decrease in patients' BMI (*t* = −1.207, *P* = 0.232), but the difference was not statistically significant.

**Conclusions:**

This study found that education based on the FCEM was associated with greater improvements in HTN self-management and blood pressure control compared with general health education among CHLC older adults with HTN.

**Clinical trial registration:**

https://www.chictr.org.cn/showproj.html?proj=194623, identifier: ChiCTR2300070940.

## 1 Introduction

Hypertension (HTN) is a critical global public health challenge. It affects an estimated 33% of adults aged 30–79 worldwide, rising sharply to 49% among those aged 50–79. Elevated systolic blood pressure (SBP) is the leading global risk factor for mortality and contributed to about 19% of all deaths in 2019. The burden of HTN disproportionately falls on low- and middle-income countries, where 78% of affected adults live. Regions like Southeast Asia bear a high absolute patient load. Despite this prevalence, control remains inadequate: only about 54% of affected individuals are diagnosed, and a mere 21% achieve effective control (1).

China bears a particularly heavy burden within this global context, being home to the world's largest hypertensive population (over 245 million). Among adults aged ≥60, prevalence reaches 58.6% (1, 2), with an uncontrolled rate as high as 85% (3). The rising prevalence is closely linked to accelerated socioeconomic development and population aging, posing a formidable challenge to health systems and necessitating focused management strategies. In response, HTN management has been incorporated into China's National Essential Public Health Service Package, a pivotal measure supported by national (e.g., Healthy China 2030) and regional (e.g., Shandong Salt Reduction Project) strategies that prioritize HTN control (4).

Despite these policy efforts, HTN management in China faces substantial challenges rooted in demographic and structural factors. For instance, among the large aging population, the crude HTN prevalence reaches 53.24%, yet only 51.35% receive treatment, with a mere 18.20% achieving control (1). Lifestyle risks persist, such as a daily salt intake over twice the WHO recommendation (5).

Systemic and behavioral barriers further complicate management: uneven primary care capacity, especially in rural areas, hampers screening and follow-up; common comorbidities like diabetes and chronic kidney disease demand integrated care; and poor patient adherence—driven by HTN's asymptomatic nature, medication costs, low health literacy, and regimen complexity—results in high discontinuation rates.

These interrelated challenges underscore the urgent need for innovative, feasible, and patient-centered strategies implementable within China's primary health care system.

The Multidimensional Health Locus of Control (MHLC) is a psychological construct reflecting individuals' beliefs about what controls their health. It comprises three dimensions: an Internal locus (IHLC, belief in personal control), a Powerful Others locus (PHLC, belief in control by providers), and a Chance locus (CHLC, belief that health is governed by fate or luck). This study focuses on patients with a predominant CHLC, defined as scoring higher on the chance dimension than on both internal and powerful others dimensions of the MHLC scale. CHLC is associated with low self-management efficacy and a higher risk of negative emotions like depression and anxiety (6). As a significant predictor of health outcomes, MHLC plays a crucial role in illness cognition, self-management, and emotional regulation.

Adherence to self-management is crucial for hypertensive control (7). However, patients with a predominant Chance Health Locus of Control (CHLC) often lack motivation for self-management due to insufficient social and family support, neglect of personal health, and low self-efficacy. Consequently, compared to patients with an internal or powerful others locus of control, those with CHLC tend to have a lower quality of life, higher complication rates, and poorer prognoses. Therefore, there is an urgent need for interventions that provide self-management education, medical resources, and sustained support specifically tailored for this vulnerable group. This study specifically focuses on older adult hypertensive patients characterized by CHLC. The Family-Centered Empowerment Model (FCEM) is a chronic disease management model that adopts a family-centered care philosophy (8). Pioneered by Dr. Fatemah Alhani, it focuses on empowering both patients and their family members to maximize the family's role in health management. Without altering routine treatment, healthcare professionals guide primary family caregivers to actively participate in the intervention process. This collaborative engagement aims to improve patient outcomes, enhance care effectiveness, and strengthen caregivers' health-related knowledge and skills (9).

The four-stage structure of FCEM directly targets the core cognitive and motivational deficits that define a chance orientation. While patients with CHLC tend to view health outcomes as uncontrollable, externally determined, and disconnected from personal action, FCEM systematically replaces these beliefs with family-anchored, action-oriented, and verifiable processes. Current hypertension management predominantly relies on patient-directed interventions. For older adults, these approaches often yield limited and unsustainable outcomes due to common limitations, including dependency on healthcare professionals (e.g., benefits of nurse-led education may diminish post-intervention), poor long-term adherence, implementation challenges, and a shortage of specialized personnel (10). In contrast, the family empowerment model offers a systemic alternative by focusing on collaborative decision-making within the family. Grounded in a four-stage framework—perceived threat identification, problem-solving, participatory education, and process/outcome evaluation (11)—it empowers patients and families to jointly manage the condition, enhancing self-efficacy and sustainable self-management (12). Supporting this, prior research demonstrates that family involvement improves medication adherence among older hypertensive patients (13).

Specifically, FCEM directly targets the cognitive and behavioral underpinnings of CHLC through its four-stage process:

Stage 1 (Perceived threat identification): Patients with CHLC tend to attribute health outcomes to luck or fate, which reduces their perception of disease threat. By guiding families to collaboratively identify personal risks of uncontrolled hypertension (e.g., stroke, heart attack), FCEM cognitively challenges the chance attribution, creating a “threat awareness” that contradicts external locus beliefs. This stage thus directly targets the fatalistic denial of personal risk that characterizes CHLC.

Stage 2 (Problem-solving): CHLC is associated with passive coping. FCEM replaces passivity with structured family problem-solving (e.g., “What can we do together if blood pressure fluctuates?”), thereby shifting perceived control from external chance to internal family action. This stage transforms the CHLC patient's expectation that “nothing can be done” into a concrete, shared task.

Stage 3 (Participatory education): Traditional education may reinforce CHLC if delivered as one-way expert advice. FCEM uses participatory dialogue where families co-construct knowledge, which directly counters the belief that health is uncontrollable. This stage fosters an internal attribution by demonstrating that learning and action produce measurable outcomes. By actively involving the patient and family in knowledge creation, this stage breaks the passive information-receiving pattern that sustains chance beliefs.

Stage 4 (Process/outcome evaluation): CHLC often persists because chance-oriented individuals do not systematically link actions to outcomes. FCEM's structured evaluation (e.g., reviewing blood pressure logs together) provides explicit evidence that family-led actions lead to improvements, thereby weakening chance attributions and strengthening internal control. This closing stage is particularly important for CHLC patients, as it supplies the empirical feedback that their belief system inherently denies.

Through this staged alignment, FCEM does not simply add family support to a CHLC patient's care plan—it systematically dismantles the cognitive, motivational, and behavioral components of chance orientation from within a family-centered process.

Building on this theoretical integration, this study adopted FCEM, combined with Traditional Chinese Medicine (TCM) principles, to deliver a structured health management program for community-dwelling older adults with hypertension characterized by CHLC. The intervention aims to improve blood pressure control and reduce patients' reliance on chance-based health attributions.

## 2 Methods

### 2.1 Study design

This study was conducted to determine the effectiveness of TCM self-management based on the FCEM for CHLC older adults with HTN in Lanzhou, China. This study comprised 2 phases. In phase 1, we structured a health education program grounded in the FCEM, which was developed through a comprehensive literature review and two rounds of Delphi expert consultation. This program is specifically designed for community-dwelling older adults with early-stage HTN.

**Figure 1:**
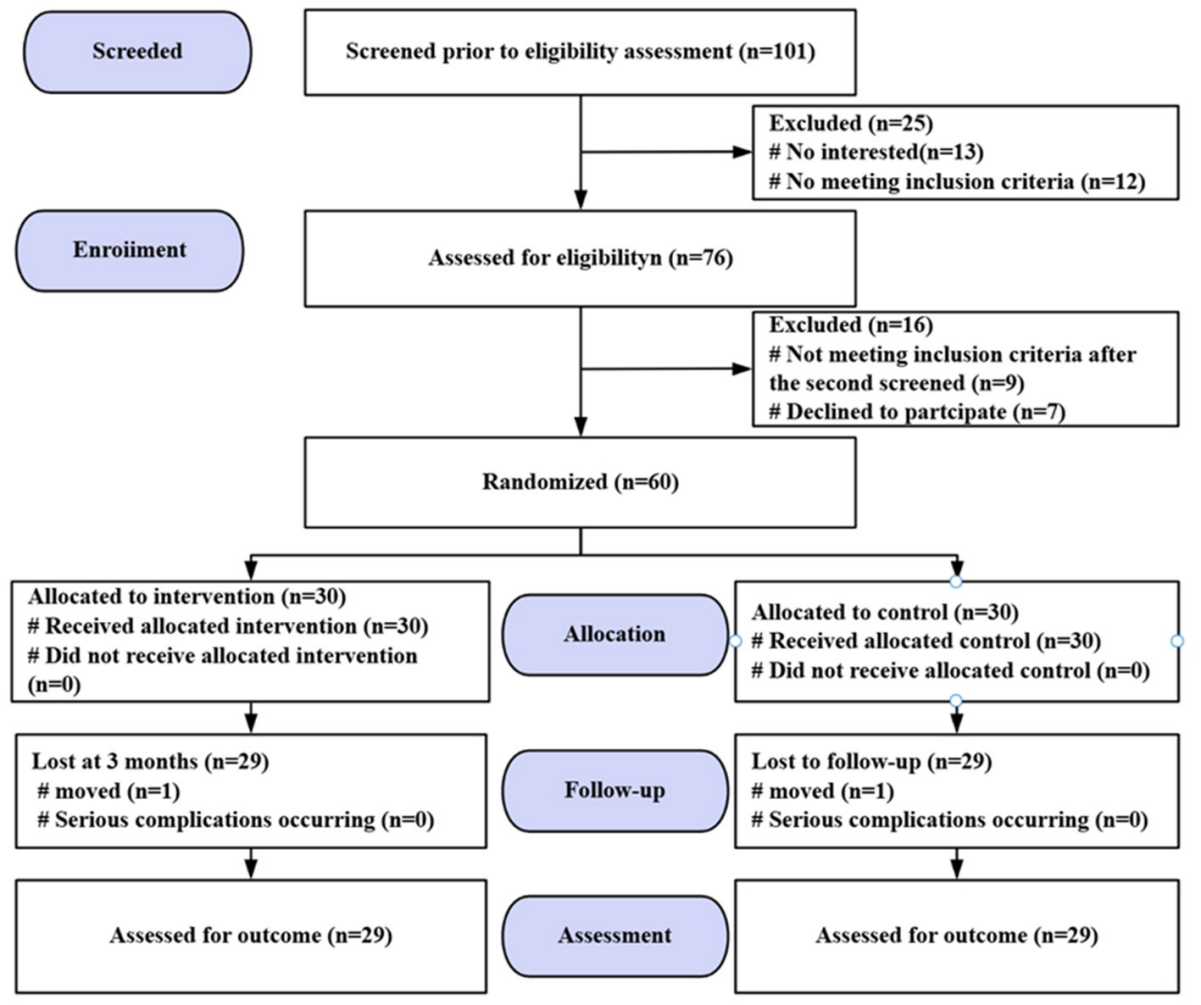
CONSORT flow diagram of participant enrollment.

In this article, we only describe the main results of the first stage (Table 1). In phase 2, we completed the implementation and evaluation components of the FCEM, executed the protocol, and analyzed the results. This constituted a 6-month two-arm parallel randomized controlled trial. Participants were recruited from communities in Lanzhou City, Gansu Province, China. A multistage sampling approach was implemented: First, a district in Lanzhou City was randomly selected through lottery sampling from Lanzhou's five districts. Subsequently, a community health center was chosen via systematic random sampling within the selected district. After obtaining informed consent, a researcher not involved in subject recruitment divided the participants into text and control groups using the randomized number table method. In addition, chronic disease management nurses at the Community Health Service Centers were contacted to participate in the study to ensure the cooperation of the participants. Figure 1 presents the participant flow diagram.

**Table 1:** Intervention programs based on the family-centered empowerment model.

Time	Stage	Content	Method
Before intervention	Perceived threats	1. Assessment of the patient's knowledge, beliefs, and behaviors through the knowledge, attitude, and behavior sections of the **HPSMBRS**. 2. Review of the patient's current year's medical report by a community-based HTN clinician to identify the patient's HTN phenotype in order to assess the patient's overall health status. (Those who have not had a medical check-up are invited by phone to the Community Health Service Center to complete the medical check-up and then view the report, or the latest one if there are multiple medical check-ups recorded in the current year.) 3. With the consent of the patient and the primary caregiver, the intervention team went to the patient's home to assess the patient's living environment in order to get a full picture of the patient's self-care, home environment, storage of medicines, and other problems.	Before the start of the intervention, each patient, after enrolment, will be assessed as a whole in the meeting room of the CHC. Participants included members of the subject intervention team, patients, and primary caregivers. After the assessment, baseline indicators were measured.
Intervening	Problem solving	1. Physicians engaged in the treatment of HTN in the community lead health education lectures once a month on topics such as the causes of HTN, the dangers of poor control, and methods of blood pressure monitoring, diet, lifestyle, exercise guidance, medication precautions, and simple, appropriate techniques for HTN. 2. Push out easy-to-understand popular science articles, cases, pictures, and videos about HTN in the WeChat group 1-2 times a week. 3. Give patients emotional care to avoid mood swings. By asking patients about their perception of the disease every 2 weeks and recording it, patients are encouraged to express their true emotions and views about the disease, while their emotions can be relieved through pentatonic therapy and silent meditation. Give patients emotional care to avoid mood swings. By asking patients about their perception of the disease every 2 weeks and recording it, patients are encouraged to express their true emotions and views about the disease, while their emotions can be relieved through pentatonic therapy and silent meditation. 4. Answer patients‘ questions promptly and assess patients' health behaviors based on their achievement of blood pressure targets at the end of the respective phase. Positive feedback is given to the patient if the target is achieved; if it is not achieved, the patient is asked about the reasons and guided to reflect and improve the program. 5. Select the patients with better adherence or obvious progress from the results of patient feedback, and the patients with better adherence will share their personal experience of self-management of disease in the next health talk, so as to motivate the patients with poorer adherence to strive to achieve their personal blood pressure control goals. 6. After the health education seminar, a community physician with more than 20 years of experience in a HTN clinic opened a clinic for guidance in order to meet the needs of the patients. Participation in the clinic is required for those with poorly controlled blood pressure in the previous phase, while other patients can participate on demand. Instruction includes: dietary modification, exercise guidance, medication guidance, personal care, and blood pressure monitoring. (Teach patients and primary carers the correct way to measure blood pressure and use the same sphygmomanometer to do so.) For those with unsatisfactory blood pressure control, measure blood pressure once a day in the morning and evening, 2-3 times each time for 7 consecutive days, and for those with satisfactory blood pressure control, measure blood pressure once a week. For patients with unstable blood pressure, i.e., high and low/ excessive differential pressure/ DBP too high and too low/ SBP too high and too low, implement other health programs (medication, blood pressure monitoring).	A total of 6 health education sessions will be held in the conference room of the CHC for approximately 45-60 minutes per session, with participants including members of the subject intervention team, patients, and their primary caregivers.
Intervening	Educational involvement	1. Participation in Knowledge Talks: Primary caregivers are required to participate in a joint health education talk with the patient once a month. 2. Involvement in medicinal cooking: In daily life, the primary caregiver participates in the cooking of meals and learns together with the patient how to prepare an appropriate diet for the hypertensive patient according to the principles of the diet. 3. (c) Learning simple appropriate techniques: carers are required to learn appropriate techniques such as acupressure, massage, and foot baths together with the patients to promote blood circulation, regulate qi and blood, and alleviate the patients' symptoms. 4. Participation in living and living: the carer works with the patient to adapt to living and living with HTN, including guidance on sleep regimen, emotional regulation, and appropriate exercise. 5. A group meeting between the patient and primary caregiver is held once a month to stimulate the potential motivation of the primary caregiver. The community doctor communicates with the patient and primary caregiver and uses questions to guide the development of the next blood pressure goal and implementation plan. 6. Patients and primary carers are invited to participate in role swaps and family case scenarios that recreate the previous stage of the problem. This promotes a level of mutual understanding.	There were a total of 6 group meetings, 1/month, with 5–6 people in each group, in the activity room for the older adults at the community health service center, for about 60–90 min each time, with participants including members of the subject intervention team, patients, and their primary caregivers.
After intervention	Effectiveness evaluation	1. Formative evaluation: Patients were asked every 2 weeks during the intervention whether they had met their weekly goals and were guided to self-assessment. 2. Process evaluation: to evaluate the satisfaction of patients and primary caregivers in the intervention group with the intervention program and to seek improvements. 3. Patients' blood pressure, BMI, and self-management behaviors were assessed at baseline, 3 months, and 6 months after the intervention.	A total of 12 sessions of 10–25 min each will be conducted through telephone contact, home visits, etc. Indicator assessments will be conducted at the CHCs.

### 2.2 Participants

Participants were selected by stratified random sampling from May 2023 to January 2024. The inclusion criteria of the participants were: (1) Permanent residents of Lanzhou City aged 65 years; (2) documented diagnosis of HTN by a tertiary-level hospital; (3) at least one caregiver (if multiple caregivers exist, the one providing the longest duration of care was enrolled); (4) Conscious, with some literacy, no language communication disorder; (5) Patients defined as “ CHLC patients ” by a dual criterion: a professional psychologist's assessment and an MHLC scale profile showing the chance dimension as the predominant one (over internal and powerful others); and (6) Voluntary participation in this study. Exclusion criteria excluded individuals who: (1) participated in other related studies. (2) exhibited severe complications impairing self-care, mobility, or communication; or (3) had documented psychiatric histories. Trial termination protocols were implemented for participants experiencing either intolerable adverse events or loss to follow-up.

### 2.3 Sample size

Sample size determination employed the formula: n_1_=n_2_ = 2[*S*(Z_α/2_+Z_β_) /δ] ^2^. where n_1_ and n_2_ are the required sample numbers for the experimental group and the control group, respectively, Typically, α = 0.05, and β = 0.10; Informed by post-intervention blood pressure data identified in the literature (14), a power analysis was conducted using G^*^Power software, Z_α/2_ = 1.96, Z_β_ = 1.282, *S* = 4.8, δ = 4.6. Consequently, the necessary sample size of each group was determined to be 23 cases; considering a 20% dropout rate, the control and experimental groups consisted of 30 cases each.

### 2.4 Intervention

During the intervention implementation, both patient groups received standardized care from physicians specializing in HTN, all of whom possessed 7 years of clinical experience. These physicians conducted weekly face-to-face consultations every Thursday to systematically address patients' inquiries about therapeutic regimens and medication. The experimental group was provided with a TCM health education program based on the FCEM. During the implementation of the trial, health education lectures were provided, and telephone follow-ups, face-to-face interviews, and home visits were conducted for the experimental group to support the implementation of the plan. According to the schedule, the implementation of health education lectures was re-planned (see Table 2).

**Table 2:** Contents of monthly health education lectures.

1	HTN: Pathophysiology and Etiology 1.1 Explanation of the causes of HTN to patients. 1.2 Explanation to patients that long-term self-management can effectively control blood pressure, and informing patients that self-management is the best way to maintain health.
2	Risks of Uncontrolled HTN and Blood Pressure Self-Monitoring 2.1 Presentation of successful self-management cases in improving blood pressure control and illustration of the systemic complications and mortality caused by poor blood pressure control through examples. 2.2 Organizing a viewing of correct blood pressure monitoring maneuvers; 2.3 Patients demonstrated blood pressure monitoring maneuvers, and researchers corrected incorrect maneuvers on the spot.
3	Dietary Management and Lifestyle Modifications for HTN 3.1 Educate patients about the importance of diet modification in patients with HTN, including appropriate food combinations and mealtime choices. 3.2 Presentation and live discussion of common misconceptions about dietary management of HTN. 3.3 Explain to patients the impact of emotions on HTN, encourage patients to express their emotions, teach them some ways to relieve their emotions, and encourage them to be optimistic about life.
4	Recognition, Prevention, and Management of Acute and Chronic Complications of HTN 4.1 Watch a short video on the impact of complications of HTN on the lives of patients when they occur, explaining the acute and chronic complications of HTN. 4.2 Teach patients the prevention, recognition, and early management of acute and chronic complications. For example, avoiding excitement and overexertion when blood pressure is high, etc.
5	Exercise Prescription and Medication Adherence in HTN 5.1 Educate patients about exercise precautions for hypertensive patients and correct misconceptions about exercise. Introduce simple, safe, and easy-to-follow exercise methods such as Walking, Taichi, and Baduanjin. 5.2 Explain to the patient the instructions and adverse effects of common medications, and ensure treatment.
6	Traditional Chinese Medicine Techniques for Hypertensive Symptoms 6.1 An individualized selection of Traditional Chinese Medicine techniques, including acupressure and moxibustion, shall be made considering the patient's constitution and preferences. Patients will receive training in the precise localization of acupoints and the correct application of massage techniques.

The intervention for the experimental group was designed as a culturally tailored, integrated health education program delivered within a family-centered empowerment framework. It comprised structured educational sessions, family collaborative conferences, regular follow-up visits, and TCM practices including Baduanjin, Tai Chi, acupoint care, and emotional nursing.

#### 2.4.1 Control group intervention

Patients in the control group received standard community-based hypertension management for 6 months. To match the contact frequency of the intervention group, the control group also received: (1) monthly telephone follow-ups (limited to checking general well-being, no health guidance); (2) weekly health information updates via WeChat group; and (3) health education lectures at the same frequency as the intervention group (covering standard hypertension knowledge, without FCEM components).

#### 2.4.2 Experimental group intervention

**Health education lectures**. Community health education lectures on HTN were conducted on the 7th of each month from May to December 2023 at local Community Health Service Centers. Before the start of each lecture, participants were notified via telephone to confirm attendance and ensure study compliance. Trained community nurses delivered the health education Lectures using Microsoft PowerPoint presentations and health education manuals. Six lectures, each lasting 60 to 90 min, were conducted between 3:00 and 5:00 p.m. during the study period to accommodate older adults' typical availability. Structured 5-min breaks were integrated every 25 min to sustain attentiveness and enhance content retention. At the end of the lectures, participants were given gifts (e.g., medicine organizer boxes) as a reward. For individuals unable to attend due to special circumstances, follow-up communications were conducted to deliver equivalent educational content covered during the lectures.

Occasional absences occurred, primarily due to unpredictable family caregiving obligations (e.g., caring for older adult parents or grandchildren).

To ensure equitable intervention exposure and fidelity for all participants in the experimental group, we implemented a standardized contingency protocol: Alternative material delivery: Absent participants received the core educational content via condensed self-study materials (handbook, videos). Remote individual follow-up: A researcher conducted a structured phone or WeChat follow-up within one week to review key points, answer questions, and reinforce learning objectives. In-person verification: During the subsequent home visit, understanding and retention of the missed content were briefly assessed to ensure parity in knowledge acquisition.

**Collaborative care conferences**. Monthly collaborative care conferences were convened for patients and their primary caregivers after the health lecture. The initial session underscored the pivotal role of caregivers to activate their latent potential and clarify their designated duties in facilitating self-management among hypertensive patients. During the conferences, targeted questions such as “How does this affect you?” “Do you want to change this?” and “What is your preferred solution?” were used to guide patients and caregivers in creating a specific implementation plan for their blood pressure goals. This included quantifying daily exercise targets using available community fitness resources and personal preferences. Primary caregivers were actively involved, for instance, by accompanying patients in exercises like Tai Chi or Baduanjin. It is equally crucial to continuously monitor and ensure that both parties fully understand the goals and the steps. Meanwhile, the conferences facilitated role-reversal exercises and simulated family scenarios for patients and their primary caregivers. These simulations recreated specific issues identified during the preceding phase, which aimed to promote mutual understanding and empathy among participants.

**Face-to-face communication and telephone follow-up**. After informed consent was obtained, participants were followed up by a 15–20-min structured telephone call between the 15th and 20th of each month by the principal investigator and community nurse. For those older adults who came to the Community Health Service Centers, face-to-face meetings were arranged with chronic disease management nurses to catch up on what was missed during the telephone follow-up visits, explained the national public health services for HTN, and helped them register for access.

**Cyclical Health Education Push**. A structured weekly content distribution plan was implemented through WeChat groups, which comprised 1-2 educational articles, case studies of uncontrolled HTN, and supplementary visual aids (images/videos).

### 2.5 Data collection

We collected baseline information such as age, gender, disease duration, and any complications. Body Mass Index (BMI), blood pressure level, MHLC scores, and self-management of HTN were measured before (T0), 3 months after (T1), and 6 months after (T2) the intervention. All physiological indices were measured by the same person using the same instrument to minimize bias. The questionnaires were completed independently by the respondents whenever possible. If they could not be completed independently, responses were obtained by the researcher after explaining them to the respondents in a non-directive manner. For missing or incorrectly completed questionnaires, the researcher checked with the respondent, and the accurately completed questionnaires were collected on the spot.

### 2.6 Ethical considerations

All participants agreed to anonymize their data. The Ethics Committee of the Affiliated Hospital of Gansu University of Traditional Chinese Medicine approved the study in December 2022: [Ethics (2022) 110] and registered in the China Clinical Trial Registry Registration No: ChiCTR2300070940.

### 2.7 Measurements

#### 2.7.1 Body mass index (BMI)

Height (m) and weight (kg) were measured to the nearest 0.1 cm and 0.1 kg, respectively, using the G-Tech scale, and BMI was calculated automatically. During measurement, participants wore light clothing and no shoes to ensure accuracy.

#### 2.7.2 Blood pressure

Participants were instructed to measure their blood pressure using a standardized, calibrated, automated electronic sphygmomanometer (Omron T-30) after a 5-minute break, with each reported value being the average of three measurements.

#### 2.7.3 Hypertension patients self-management behavior rating scale (HPSMBRS)

The scale developed by Wallston (6) in 1978 was used for measurement. The scale consists of three dimensions: internal health locus of control, powerful others health locus of control, and chance health locus of control, with six items per dimension, totaling 18 items. Specifically, items 1–6 assess the internal dimension, items 7–12 assess the powerful others dimension, and items 13–18 assess the chance dimension. Each item is rated on a 6-point Likert scale (1 = strongly disagree, 2 = disagree, 3 = slightly disagree, 4 = slightly agree, 5 = agree, 6 = strongly agree). The three subscales are scored separately, and the dimension with the highest score determines the patient's predominant locus of control orientation; a higher score indicates a stronger tendency toward that specific locus of control. The scale demonstrated good reliability and validity, with Cronbach's α coefficients of 0.872, 0.925, and 0.803 for the three subscales, respectively. This scale measures participants' perceptions of health control over a defined retrospective period, which provides a relatively stable snapshot of their orientation.

#### 2.7.4 HTN patients self-management behavior rating scale (HPSMBRS)

The scale was compiled by Chinese scholars Zhao et al. (15). It included six dimensions: medication management, disease monitoring, diet management, exercise management, rest and activity management, and emotion management, with a total of 33 items. A 5-point Likert scale was used, ranging from 1(never) to 5 (often). The score ranges from 33 to 165, with higher scores indicating better self-management behavior. The Cronbach's alpha coefficient of the scale is 0.914, and the content validity is 0.910.

To enable cross-dimensional score comparison, the raw scores of the two scales were converted into standardized scores. Standardized score = (actual full score for each entry/full score for each entry) £ 100. Based on the standardized scores, self-management levels were categorized into three grades: low (< 60), medium (60~80), and high (>80).

### 2.8 Data analysis

Data entry was performed by 2 researchers using EpiData 3.1, and data processing was performed using IBM SPSS 26.0 statistical software. Statisticians were blinded to the study group. α = 0.05, *P* < 0.05 indicated a statistically significant difference. All study participants were subjected to analysis, and any missing values were subsequently filled in using multiple interpolations for the six participants who were lost to follow-up. Measurements were first subjected to the Kolmogorov-Smirnov test to assess normality, with conformity to normality described by mean and standard deviation, and nonconformity to normality described by mean and interquartile range. Bartlett's chi-squared test of variance was then performed to test the assumption of chi-squared variance. The statistical descriptions of the count data were performed using a combination of frequency, component ratios, independent samples *t*-tests for between-group comparisons, and additionally analyses of between-group, time, and time-group interactions employing repeated-measures ANOVA.

## 3 Results

### 3.1 Baseline characteristics

Analysis of demographic characteristics indicated that the 58 enrolled patients had an age range of 65 to 89 years. The cohort was equally distributed by gender (50% each), and the majority were married or cohabiting (79.3%). In terms of education, 43.10% had attained a junior high school level or below. The most frequent monthly household income per capita was less than 1,000 yuan (29.30%), and employee medical insurance was the primary payment method (51.72%). Regarding disease duration, the most common category was 11–15 years (29.31%), while 60.34% of patients reported no family history of the disease. Statistical comparisons confirmed that there were no significant differences in these baseline characteristics between the two patient groups (*P* > 0.05). For detailed data, please refer to Tables 3–5.

**Table 3:** Comparing basic patient characteristics across groups (*N* = 58).

Characteristics		The experimental group (*n* = 29)	The control group (*n* = 29)	Test statistic	*P*
		*n* (%)	*n* (%)		
Gender	Male	13 (44.80)	16 (55.20)	0.360	0.548
	Female	16 (55.20)	13 (44.80)		
Age(years)	60	17 (58.62)	18 (62.07)	1.732	0.188
	75	12 (41.38)	11 (37.93)		
Marital status	Married/Widowed	20 (69.00)	26 (89.70)	3.783	0.052
	Divorced/widowhood/solitary	9 (31.00)	3 (10.30)		
Education levels	Junior high school and below	13 (44.80)	12 (41.40)	4.185^*^	0.125
	High School/Technical Secondary School	9 (31.00)	15 (51.70)		
	Bachelor's degree or post-secondary degree	7 (24.10)	2 (6.90)		
Monthly per capita household income(yuan)	< 1,000	9 (31.02)	8 (27.60)	2.752^*^	0.435
	1,000 1,999	8 (27.60)	11 (37.90)		
	2,000 2,999	6 (20.69)	8 (27.60)		
	≥3,000	6 (20.69)	2 (6.90)		
Means of coverage of medical expenses	Employee medical insurance	14 (48.30)	16 (55.20)	2.178^*^	0.595
	Resident health insurance	7 (24.10)	8 (27.60)		
	NCMS	7 (24.10)	3 (10.30)		
	Others	1 (3.50)	2 (6.90)		
Duration of illness	≤ 5	10 (34.40)	3 (10.30)	4.109	0.250
	6 10	7 (24.20)	9 (31.00)		
	11 15	6 (20.70)	11 (38.00)		
	≥16	6 (20.70)	6 (20.70)		
Family history	Yes	12 (41.40)	11 (37.90)	0.072	0.788
	No	17 (58.60)	18 (62.10)	

^*^P < 0.05.

**Table 4:** Comparison of physiological measures by group (*N* = 58).

Indicator	The experimental group	The control group	Test statistic	*P*
BMI (Kg/m2)	24.50 ± 3.73	24.89 ± 3.04	0.427	0.671
SBP (mmHg)	145.83 ± 8.64	146.14 ± 3.70	0.178	0.860
DBP (mmHg)	86.59 ± 4.83	88.90 ± 5.68	1.669	0.101

**Table 5:** Comparison of basic health locus of control and self-management scores between the two study groups (*N* = 58).

Measurement indicator		The experimental group (*n* = 29)	The control group (*n* = 29)	Test statistic	*P*
MHLC					
	IHLC score	21.14 ± 1.83	21.28 ± 1.51	−0.313	0.755
	PHLC score	19.34 ± 2.58	19.72 ± 2.31	−0.589	0.558
	CHLC score	23.86 ± 3.17	23.52 ± 2.17	0.484	0.630
Self-management score		94.86 ± 9.14	95.59 ± 7.81	−0.324	0.747

### 3.2 The Impact of the FCEM on physiological indicators, health locus of control, and self-Management among patients in two groups

Following a three- and six-month intervention period, no statistically significant differences were observed in BMI between the two groups (*P* > 0.05). Meanwhile, statistically significant differences were noted in SBP, DBP, HPSMBRS points, and between the two groups after three and six months of intervention (*P* < 0.05). See Table 6. In the three-month intervention period, assessment of the Health Locus of Control scales at the 3-month intervention point revealed significance solely in the internal control dimension. In contrast, the 6-month assessment yielded statistically significant outcomes across all three dimensions. See Table 7.

**Table 6:** Comparison of SBP, DBP, HPSMBRS scores and BMI between the two groups at different time points (*N* = 58).

Time	Variable	Intervention group	Control group	*t*	*P*
T0	SBP	145.83 ± 8.64	146.14 ± 3.70	0.178	0.860
	DBP	86.59 ± 4.83	88.90 ± 5.68	1.669	0.101
	HPSMBRS scores	94.86 ± 9.14	95.59 ± 7.81	−0.324	0.747
	BMI	24.50 ± 3.73	24.89 ± 3.04	0.427	0.671
T1	SBP	141.79 ± 7.67	145.93 ± 3.76	−2.609	0.012^*^
	DBP	82.03 ± 6.65	88.38 ± 5.38	−3.997	< 0.001^*^
	HPSMBRS scores	103.55 ± 8.58	97.69 ± 6.61	2.914	0.005^*^
	BMI	24.10 ± 2.70	24.88 ± 3.05	−1.028	0.309
T2	SBP	139.31 ± 7.96	144.66 ± 3.40	−3.325	0.002^*^
	DBP	80.14 ± 6.20	87.69 ± 4.99	−5.110	< 0.001^*^
	HPSMBRS scores	109.14 ± 10.60	99.00 ± 6.41	4.406	< 0.001^*^
	BMI	23.98 ± 2.67	24.88 ± 3.04	−1.207	0.232

^*^P < 0.05.

**Table 7:** Comparison of IHLC, PHLC, and CHLC between the two groups at different time points (*N* = 58).

Time	Variable	Intervention group	Control group	*t*	*P*
T0	IHLC	21.14 ± 1.83	21.28 ± 1.51	−0.313	0.755
	PHLC	19.34 ± 2.58	19.72 ± 2.31	−0.589	0.558
	CHLC	23.86 ± 3.17	23.52 ± 2.17	0.484	0.630
T1	IHLC	23.24 ± 1.68	23.24 ± 1.68	5.629	< 0.001^*^
	PHLC	21.10 ± 2.39	20.03 ± 2.18	1.778	0.081
	CHLC	21.93 ± 2.58	22.69 ± 2.51	−1.136	0.261
T2	IHLC	25.21 ± 1.86	20.55 ± 2.29	8.493	< 0.001^*^
	PHLC	23.34 ± 1.90	21.10 ± 2.27	4.078	< 0.001^*^
	CHLC	19.21 ± 3.02	22.70 ± 2.51	−4.781	< 0.001^*^

^*^P < 0.05.

The repeated-measures ANOVA demonstrated that SBP, DBP, and HPSMBRS points exhibited statistically significant results in the dimensions of time effect (*P* < 0.001, *P* < 0.001, and *P* < 0.001), intergroup effect (*P* = 0.046, P < 0.001, and P =0.014), and time-group interactions effect(*P* < 0.001, *P* =0.031, and *P* < 0.001). Similarly, IHLC and CHLC points demonstrated statistically significant differences in the dimensions time effect (*P* < 0.001, *P* < 0.001), intergroup effect(*P* < 0.001, *P* =0.044), and time-group interactions effect (*P* < 0.001, *P* < 0.001). Whereas PHLC was statistically different only in the dimensions of time effect (*P* < 0.001) and time-group interactions effect (*P* < 0.001). Besides, the BMI did not exhibit statistically significant differences in any dimensions. See Tables 8, 9.

**Table 8:** Repeated measures ANOVA for SBP, DBP, HPSMBRS scores, and BMI in both groups (*N* = 58).

Variable	Intergroup		Time		Time-group interaction	
	*F*	*P*	*F*	*P*	*F*	*P*
SBP	4.159	0.046^*^	68.992	< 0.001^*^	29.755	< 0.001^*^
DBP	20.665	< 0.001^*^	31.355	< 0.001^*^	3.016	0.031^*^
HPSMBRS scores	6.434	0.014^*^	71.209	< 0.001^*^	26.820	< 0.001^*^
BMI	0.847	0.361	0.587	0.558	0.587	0.558

^*^P < 0.05.

**Table 9:** Repeated measures ANOVA for IHLC, PHLC, and CHLC in both groups (*N* = 58).

Variable	Intergroup		Time		Time-group interaction	
	*F*	*P*	*F*	*P*	*F*	*P*
IHLC	38.674	< 0.001^*^	57.670	< 0.001^*^	26.247	< 0.001^*^
PHLC	1.198	0.278	21.705	< 0.001^*^	20.071	< 0.001^*^
CHLC	4.239	0.044^*^	83.970	< 0.001^*^	40.924	< 0.001^*^

^*^P < 0.05.

## 4 Discussion

A six-month community health education program was developed based on family-centered empowerment theory to improve self-management and reduce blood pressure in CHLC older adult patients with HTN. While the value of family empowerment in chronic disease management is established in prior research (16), scant intervention resources have been tailored for individuals exhibiting the specific psychological profile of a CHLC. For CHLC older adults with HTN, it is vital to provide them with reliable caregiver support and HTN-related knowledge, to mitigate their chance dimension scores and foster a psychological shift toward internal control (17).

Health education is a cornerstone, and it is instrumental in enhancing patients' capabilities to self-manage HTN. Previous studies have indicated that community-dwelling older adults with HTN who exhibit a high CHLC typically demonstrate poorer self-management capabilities and lower intrinsic motivation. Consequently, these patients often require targeted external interventions to shift their health beliefs (i.e., reduce their CHLC scores) and, in turn, enhance their self-management abilities.

In our study, we built upon previous findings indicating that CHLC older adult patients with HTN need structured interventions and require enhanced family support opportunities to facilitate behavioral and attitudinal modification (18). Therefore, the intervention program was further enhanced by incorporating group meetings and role-reversal exercises, enabling patients to implement personalized intervention plans with active family supervision and support. This was complemented by group sessions that delivered practical knowledge and monitoring techniques. Furthermore, integrating tailored TCM practices—such as acupressure and Baduanjin—into a family empowerment framework not only fosters a sense of autonomy and disease control among CHLC patients but also actively involves family members in assisting and supervising the intervention process.

This synergy between family support and structured education, surpassing the effect of traditional health management programs alone, empowers patients to implement and sustain self-management behaviors more effectively, thereby achieving clinically significant improvements in blood pressure regulation.

Previous studies have shown that HTN has a great influence on cardiovascular disease (19). A review found that each 10 mmHg decrease in SBP (mean SBP of 120–124 mmHg) was associated with a 29 % reduction in cardiovascular disease and 27 % reduction in all-cause mortality compared with a mean SBP of 130–134 mmHg (20).

In our study, the six-month comprehensive health education program demonstrated significant effectiveness in CHLC patients with HTN. At the 3-month follow-up, systolic blood pressure and diastolic blood pressure (DBP) were 141.79 ± 7.67 mmHg and 82.03 ± 6.65 mmHg, respectively. By the 6-month follow-up, these values had decreased to 139.31 ± 7.96 mmHg and 80.14 ± 6.20 mmHg, respectively. Following the intervention, both SBP and DBP values showed statistically significant reductions in CHLC patients with HTN across the study groups(*P* < 0.05). The result met the standards of *the 2023 Chinese Guideline for the Management of HTN in the Older adult* (21). This finding demonstrates that a health education program based on the TCM FCEM effectively supports blood pressure management and helps maintain levels within recommended target ranges among community-dwelling older adult hypertensive patients with a CHLC.

A community-based, cluster-randomized controlled trial conducted in Nepal demonstrated that multiple health education sessions, complemented by regular household visits, can effectively enhance HTN-related knowledge and reduce blood pressure among patients with uncontrolled HTN at the community level (22). Sixty-one older adult hypertensive patients were randomly assigned to either an experimental group receiving a family empowerment-based intervention or a control group receiving routine care. The results indicated a statistically significant difference in blood pressure between the two groups (*P* < 0.05) (14). These findings are consistent with our study.

The significant between-group difference in blood pressure reduction may be explained by the core component of the intervention: the application of the family empowerment model. We hypothesize that this model effectively mobilized familial resources, providing participants in the experimental group with substantially greater practical and motivational support for medication taking, diet, exercise, and symptom tracking than was available to the control group. This augmented support system likely served as a critical facilitator, enhancing patients' capacity and commitment to daily self-management. Existing research provides evidence for this strategy. In a self-controlled trial conducted by Zhang et al. (23), 36 low-income hypertensive patients received a family empowerment model-based health intervention. The results indicated that interventions rooted in this model significantly reduced blood pressure in the study population. A cluster randomized controlled trial confirmed the efficacy of the family empowerment model in reducing blood pressure levels in patients with HTN (14).

Second, the implementation of a family empowerment model, which is centered on developing personalized health plans, serves to actively engage patients in the management process and bolster their self-management competencies. Specifically for patients predisposed to a CHLC, this model enhances dyadic communication with primary caregivers, thereby promoting the practical application of health plans. Furthermore, the social and emotional support provided within this framework improves patients' psychological wellbeing, a change that is reflected in elevated scores on the PHLC dimension and reduced scores on the CHLC dimension of health locus of control scales.

A Scoping Review showed that education involving families of the older adult with HTN can affect a decrease in blood pressure, an increase in self-efficacy and good behavioral change, an increase in self-esteem, improved life quality, and family empowerment (24).

Finally, the intervention strengthened the communication network among patients, their primary caregivers, and healthcare providers through regular home visits. It incorporated TCM techniques, such as massage and moxibustion, to regulate physiological functions. Furthermore, by instructing patients in acupoint self-massage, the intervention empowered them to actively participate in their own care, enhancing their sense of self-efficacy. Collectively, these components increased perceived social support, improved mental wellbeing, mitigated feelings of helplessness, and ultimately facilitated a shift in health locus of control from external to internal attributions among community-dwelling older hypertensive patients with a CHLC.

Additionally, there was an interaction effect on SBP and DBP, indicating that the intervention effect on SBP and DBP would gradually increase over time in the presence of the intervention.

This suggests that patients with an external health locus of control may benefit from sustained family support. In contrast, those in the control group who held the same CHLC faced a dual deficit: limited access to structured health education and a lack of targeted familial support. This combination likely impeded their progress in shifting toward a more internalized locus of control and, consequently, their adoption of recommended health behaviors.

This study underscores the pivotal role of the family environment in managing HTN among community-dwelling older adults. By applying a FCEM, it repositions the primary caregiver from a supervisor to a collaborative partner, thereby fostering greater adherence to health plans and attainment of blood pressure control goals. Diverging from prior patient-centric approaches, this intervention treats the patient-caregiver dyad as an integrated unit. Granting shared responsibility and autonomy enhances proactive engagement in disease management. Furthermore, guided by the holistic principles of TCM, the intervention transcends mere blood pressure monitoring. It formulates personalized management plans based on the patient's comprehensive health profile. Through systematic education, both caregivers and patients are equipped to implement these plans effectively, leading to sustainable blood pressure control.

Enhanced self-management skills in hypertensive patients contribute significantly to improved blood pressure control, reduced cardiovascular risk, and enhanced quality of life (25). Approximately 50 % of cardiovascular disease risk is attributable to modifiable lifestyle (26). Self-monitoring of blood pressure has been recognized as a potential modifiable risk factor in the progression of cardiovascular disease (27) and in reducing the burden of cardiovascular disease (28).

Self-management education for Chronic illness has become an integral part of primary care (29). Enhancing self-management skills in hypertensive patients significantly improves blood pressure control, lowers the risk of cardiovascular complications, and promotes overall quality of life (25). Additionally, effective self-management practices facilitate early disease detection and control, decrease the incidence of complications, and mitigate the associated financial burden on patients and families (30). Enhancing self-management capacity in hypertensive patients is fundamental to effective personal health management. Evidence demonstrates that regular self-monitoring of blood pressure significantly strengthens patients' ability to manage their condition (31).

Our intervention encouraged proactive engagement in blood pressure monitoring and management among both patients and primary caregivers by promoting regular self-measurement of blood pressure and systematic documentation of self-management activities. During this process, both patients and caregivers showed increased engagement. Through ongoing tracking of objective vital signs such as blood pressure, patients were able to monitor health parameter fluctuations in real time and to observe the association between their behavioral adjustments and changes in these parameters. Patients' self-management capacity also improved following the intervention. The family plays a critical role in shaping patient health outcomes, as caregivers' behaviors and attitudes significantly influence both health status and self-management capabilities (32). Viewing patients and caregivers as an integrated unit allows patients to benefit from a more robust support system. With empathy-based emotional support from family caregivers, patients' self-management capacity gradually improved. Additionally, TCM health education interventions—such as guidance on daily living care—helped modify lifestyle and behavioral factors underlying disease development. After the implementation of these integrated approaches, patients adopted more active roles in managing their personal health, and their overall self-management abilities also improved.

The results of this study indicate that the self-management scores of patients in the experimental group gradually increased, rising from (94.86 ± 9.14) points before intervention to (103.55 ± 8.58) points. At the conclusion of the intervention, the final self-management score for the experimental group reached (109.14 ± 10.60) points, demonstrating a significant difference (*P* < 0.001).

The findings of this study showed that a community health education program based on the FCEM was more effective than traditional health education in enhancing the self-management of CHLC older adults with HTN.

A clinical trial investigating home care for older adult patients after total knee arthroplasty found that those receiving an FCEM intervention showed significantly greater improvement in self-management capacity compared to the conventional care group (33). Another interventional study involving patients with type 2 diabetes demonstrated that the implementation of a family-centered empowerment program significantly enhances self-management capabilities in this population. (*P* < 0.001) (34). The results of the aforementioned studies align with the outcomes observed in the present investigation.

Research indicates that this intervention was associated with elevated older adults' self-esteem and self-worth through a structured pathway that involved raising health awareness and positive attitudes, actively engaging family members, and leveraging family skills under community nurse guidance. Furthermore, it facilitates the transfer of health knowledge and self-care skills from older adults to their families. In this study, this collaborative, skill-sharing process was observed to occur alongside enhanced self-efficacy and improved disease management capabilities.

Another study conducted by Cheraghi et al. also demonstrated that family-centered care can improve the management behaviors of diabetic patients and their caregivers in the realms of “blood glucose testing,” “insulin therapy,” “meal plan,” and “physical activity” (35). Amani et al. (34) also argued that a family empowerment model has a positive impact on improving the family's diet management ability, motivating the patient to do regular exercise, and use the health care facilities. In line with these findings, the present intervention was observed to be associated with strengthening the spirit of independence, accountability, self-control, identifying opportunities, motivation, improving skills, and being self-efficient. However, our study design does not establish a causal relationship between self-efficacy and disease management outcomes; this possibility should be examined in future research.

At the three-month intervention point, patients‘ internal control dimension scores in the experimental group increased, and this change reached statistical significance. Meanwhile, CHLC dimension scores had registered a marked decline, albeit not statistically significant. By six months, patients in the experimental group showed a further increase in their IHLC dimension score to (25.21 ± 1.86) points, while their CHLC dimension score decreased from the initial (23.86 ± 3.17) points to (19.21 ± 3.02) points. These results attained statistical significance. These findings suggest that for CHLC patients—those ascribing health outcomes to fortune—the protracted application of the FCEM was associated with improvements in patients' psychological state. Specifically, the observed changes were consistent with a shift toward a more perceptible internal locus of control and away from the conviction that health is dictated by luck.

We propose a two-phase mechanism to explain how the FCEM shifts patients from a chance-oriented locus of control toward a more internal orientation.

Phase 1 (0–3 months): Belief destabilization. During the initial three months, the FCEM challenges existing chance attributions through threat identification and collaborative problem-solving. Through family collaborative conferences, patients and caregivers jointly identify personal risks of uncontrolled hypertension. This process cognitively challenges the fatalistic belief that health outcomes are determined by luck or fate, creating a state of “threat awareness.” However, at this stage, patients have not yet developed stable internal beliefs; This explains why, at the 3-month follow-up, IHLC scores showed a significant increase (*P* < 0.001), while CHLC scores showed a decline that did not reach statistical significance (*P* = 0.261). Patients had begun to doubt their chance beliefs but had not yet internalized a sense of personal control.

Phase 2 (3–6 months): Belief internalization. With continued exposure to participatory education and structured outcome evaluation, patients learn to link their actions directly to clinical outcomes. Through the structured follow-up components (monthly telephone calls, weekly health information updates via WeChat group, and periodic face-to-face meetings), patients and families repeatedly review blood pressure logs and link daily behaviors to blood pressure changes. This repeated “action–outcome coupling” provides empirical evidence that personal and family-led actions produce measurable health improvements. Over time, this feedback loop gradually weakens chance attributions and strengthens internal control beliefs, with both changes reaching statistical significance by the 6-month follow-up (*P* < 0.001 for both IHLC and CHLC).

From the perspective of self-determination theory (SDT), this two-phase mechanism can be understood as a process of satisfying basic psychological needs for competence and relatedness. A large-scale meta-analysis of 184 SDT-based studies in health contexts confirmed that satisfaction of basic psychological needs—particularly competence and relatedness—is associated with better mental and physical health outcomes, with effect sizes ranging from small to large (ρ = 0.22–0.62) (36).

In our intervention, the need for relatedness was addressed through family collaborative conferences, where patients and primary caregivers jointly identified health risks, set goals, and shared successes. The need for competence was addressed through successful blood pressure monitoring experiences, where patients could directly observe the results of their actions. SDT predicts that need satisfaction can lead to behavioral change even before full internalization of beliefs—a prediction consistent with our temporal data: at 3 months, IHLC scores in the intervention group were significantly higher than those in the control group (*P* < 0.001), while CHLC scores showed no significant between-group difference (*P* = 0.261). The early improvement in internal locus of control coincides with the establishment of family collaborative support (relatedness satisfaction) (37). The delayed significant improvement in CHLC at 6 months (*P* < 0.001) may reflect the time needed for competence need satisfaction to accumulate through repeated successful self-monitoring experiences.

Complementing this, habit formation theory provides additional insight into the temporal pattern of our results (38). This theory posits that behaviors and associated beliefs become automatic through repetition in a stable context. The strength of a habit is determined by the number of times a behavior is repeated in a stable context, not by the strength of one's beliefs or intentions. Research has demonstrated that successful habit formation for simple health behaviors requires a median of 59 days to reach peak automaticity, and that repeated plan enactment is the key predictor of habit formation (39). In our intervention, the 6-month repetitive nature of the program—weekly health information updates via WeChat group, monthly collaborative care conferences, and daily blood pressure monitoring—provided precisely the stable context for repetition.

Notably, the significant time-group interaction effects for SBP, DBP, HPSMBRS scores, IHLC, PHLC, and CHLC (all *P* < 0.05) indicate that the intervention effects gradually increased over time, which is consistent with the time course of habit formation (39). The longer the intervention was sustained, the stronger the effects became. This supports the notion that reducing deep-seated fatalistic beliefs (CHLC) and consolidating internal control beliefs (IHLC) requires sustained repetition over time rather than short-term exposure.

SDT explains the motivational mechanisms—why need satisfaction drives belief change—while habit formation theory explains the temporal mechanisms—why sustained repetition produces stronger effects. This integrated understanding is particularly relevant for CHLC patients, who typically lack intrinsic motivation and hold fatalistic health beliefs (36, 40). For such individuals, the pathway to improved health beliefs may not be direct; rather, it requires first satisfying the need for relatedness through family support (driving early IHLC improvement), then accumulating competence through repeated success experiences (driving later CHLC reduction), and finally consolidating both through habit formation. Our data demonstrate that this multi-mechanism process takes approximately 6 months to achieve full effects across all three dimensions of health locus of control.

Importantly, this two-phase mechanism helps distinguish between alternative explanations. The early significant improvement in IHLC at 3 months (*P* < 0.001) suggests that internal control beliefs can be enhanced relatively quickly through family collaborative support (relatedness need satisfaction). However, the delayed significant reduction in CHLC – which did not reach between-group significance until 6 months (*P* < 0.001) – indicates that reducing deep-seated fatalistic beliefs requires more sustained, structured opportunities to link actions to outcomes. Thus, the temporal pattern of our data supports the interpretation that different mechanisms operate on different timelines: relatedness-driven belief change occurs earlier, while competence-driven and habit-driven belief change requires more time to accumulate.

A health education program grounded in the health locus of control theory significantly improved overall locus of control profiles in individuals with type 2 diabetes. However, it was specifically the internal locus of control dimension that showed sustained enhancement following program completion (41). Meanwhile, a health education intervention based on the health locus of control theory, conducted among 180 patients with type 2 diabetes by Ebadi et al. (42), demonstrated that after 2–3 months, participants' internal locus of control scores increased significantly, while their PHLC and CHLC scores decreased. These findings diverge from those of the present study, which employed an FCEM explicitly designed to strengthen the patient–primary caregiver dyad. In our cohort of patients with high CHLC scores, this approach resulted in a marked reduction in CHLC dimension scores and a concurrent increase in scores related to PHLC. Furthermore, the baseline CHLC dimension scores in our participants were substantially higher than those reported in the cited literature. This combination of a more intensive intervention and a higher-risk baseline may account for the more pronounced decline in opportunity dimension scores observed in our study.

This divergence highlights how intervention effects are shaped by population-specific characteristics. Our participants, defined by high baseline CHLC, were marked by significant helplessness. In response to this characteristic, the intervention did not immediately advocate for internal control. This progression is consistent with stage-based models of behavior change, which posit that individuals may move through intermediate stages—from precontemplation to contemplation to action—before achieving sustained internalization of health beliefs (43, 44).

Instead, by building a stable and supportive family environment, it served as a crucial stepping stone, redirecting the patients' locus from the unpredictability of “Chance” to the reliability of “Powerful Others” as a form of controllable external support. In this framework, the “Powerful Others” (family members) represent a proximal source of support, distinct from the distal medical establishment. The resulting dependency may foster an interactive and synergistic partnership. From this perspective, what the scale measures as an elevated “powerful others health locus of control” can be interpreted as a vital and positive step away from fatalistic Chance beliefs. This interpretation suggests that for individuals with very low self-efficacy, the pathway to improved health beliefs may not be direct, but can be conceptually bridged by establishing a state of “collaborative external control” as a critical stepping stone.

Our intervention actively engaged both patients and primary caregivers in the health management process, but did not produce significant changes in the PHLC dimension. While a certain degree of reliance on this dimension may enhance treatment adherence, excessive dependence on external guidance can compromise patients' autonomy and sense of responsibility in managing their health. Importantly, the absence of a significant increase in PHLC does not undermine our conclusion that self-management behaviors improved. As argued above, behavioral improvement and belief internalization are distinct constructs; the former can occur without the latter, particularly in the context of structured family support.

The FCEM fundamentally aims to cultivate patients‘ and families' intrinsic belief in their ability to influence health outcomes. In this study, as participants develop greater self-efficacy through this process, their need for external validation was observed to decrease correspondingly.

Notably, previous research has indicated that for individuals with a CHLC, self-empowerment alone is insufficient to induce changes in their health locus of control profile (45).

Viewed in light of this finding, the demonstrable need for external support in managing opportunistic patients aligns precisely with the core strength of FCEM: its systematic provision of structured, sustainable support within the family unit. This alignment suggests the model's feasibility and theoretical soundness in this context.

The risk of cardiovascular disease in hypertensive patients exhibits a positive correlation with BMI (46). This association is largely attributable to the fact that obesity contributes to elevated blood pressure through multiple pathophysiological mechanisms, thereby rendering blood pressure management more challenging and complex in obese individuals. Consequently, BMI reduction is widely recommended as a cornerstone of HTN management. Nevertheless, the present study failed to demonstrate a significant improvement in BMI among older adult HTN patients with CHLC following the intervention.

This null finding points to an important distinction that the FCEM with TCM health education may preferentially influence adherence-related behaviors (e.g., medication adherence, regular blood pressure monitoring) because these are more directly controllable by patients and families, rather than complex lifestyle modifications (e.g., dietary restructuring, sustained physical activity) that require sustained effort and environmental changes.

The inability of the FCEM to significantly impact BMI in hypertensive patients, as observed in this study and supported by others (47), creates a notable discrepancy with the BMI control efficacy reported by Zhang et al. (23) and the absence of such an effect in the current investigation.

Long-term habit change is fundamental to BMI reduction. The lack of a statistically significant change in BMI may be attributed to several factors. First, interventions centered on health education may have a more limited direct effect on energy balance compared to their stronger influence on medical behavior adherence, such as medication compliance. This further supports the above distinction: the present intervention is more effective in improving adherence-related behaviors that are more directly controllable by patients and families.

Second, older adult patients' preference for routine and reduced adaptive capacity present substantial barriers to lifestyle change (48).

Third, physiological changes associated with aging—including a decline in basal metabolic rate and reduced capacity for physical activity—can slow the pace of weight change. Supporting this, existing literature (49)indicates that a lower metabolic rate in older adults impedes their ability to achieve and sustain an optimal BMI, even with participation in structured health education.

Furthermore, the follow-up duration in this study may have been insufficient to capture long-term weight dynamics in older adults. Additionally, it is important to note that BMI, as a composite metric, does not differentiate between changes in fat mass and muscle mass. Future research incorporating body composition analysis would enable a more nuanced evaluation of intervention effects on body composition.

Finally, variations in patients' commitment to lifestyle modifications may also stem from their prioritization of blood pressure control over weight management as a primary health objective.

In summary, effective weight management for older adults with HTN likely necessitates the design of higher-intensity, longer-duration, and multidisciplinary programs that integrate personalized nutritional guidance and structured exercise support.

To directly address the cultural specificity and generalizability of our empowerment model, we conducted a cross-cultural comparative analysis of key studies, guided by the contrasting cases of the United States, Iran, and China.

Evidence indicates that in the United States, the FCEM improves patient health outcomes by engaging “family supporters.” (32)These individuals, encompassing family or friends irrespective of co-residence, facilitate greater patient involvement in healthcare and foster sustained adherence to care plans, which mitigates the risk of complications. The mechanism for this integration involves community-based extension services—such as coaching sessions, telephone reminders, and visit summaries—which optimize familial support within existing healthcare frameworks. Consequently, this approach augments patients' self-management competencies and their confidence in effectively utilizing healthcare resources, illustrating a model of structured familial support embedded within an individualistic healthcare paradigm.

Within Iranian society, the family is deeply valued as the cornerstone of support and care (17). Accordingly, the FCEM model in Iran has been localized to follow a clear, four-step educational process. At its core, this process aims to systematically instill health management knowledge, skills, and a sense of responsibility within the family. The primary caregiver is always a permanent family member living with the patient. In practice, communities host the program, but professional healthcare staff (like community nurses) have little involvement (14). In fact, studies show nurses are not present during group discussions; sessions are run entirely by patients and their caregivers, meaning professionals do not guide daily care decisions at home. The model's ultimate goal is to make the family itself a key part of the healthcare system, building its self-care capacity to work alongside social services. This approach is fundamentally different from the family empowerment model used in the United States.

Meanwhile, in China, socioeconomic development and declining fertility rates have rendered both families and medical institutions responsible for caring for older adult patients with chronic diseases (50). This study not only adopts the structured four-step educational process of the Iranian model but also retains the supervisory, guiding, and educational roles played by the community in patient care. Under the guidance of community doctors and nurses, the intervention employs methods such as telephone follow-ups, home visits, group discussions, and role-playing. These activities aim to enhance the capabilities of primary caregivers, improve patients' self-management skills, and address their psychological wellbeing. The goal is to effectively bridge families and communities, harnessing both familial and societal support. Thus, this intervention model represents an intermediate approach between those of the United States and Iran.

This study confirms the efficacy of the empowerment model in this unique subgroup. Furthermore, the implementation of TCM health education within an FCEM enhances patients' capabilities and confidence through specific components such as role-playing exercises in group sessions and collaborative goal-setting with family support. This approach fostered a sense of involvement and autonomy in managing their health interventions. This integrated strategy presents a novel, patient-centered approach to managing HTN in community settings.

In summary, this study demonstrates that FCEM combined with TCM health education significantly improves self-management behaviors and blood pressure control in community-dwelling older adult hypertensive patients with CHLC. This intervention shifted patients' health beliefs from an external toward a more internal orientation, accompanied by substantial behavioral improvements—a result that holds clinical significance for this population. We suggest that for older adults with CHLC, behavioral change may precede and potentially facilitate subsequent belief internalization.

## 5 Limitations

This study has several limitations that point to clear priorities for future research. The six-month assessment period was likely insufficient to capture the full, long-term effects of the intervention on both beliefs and physiological outcomes like BMI. Future work should, therefore, incorporate extended follow-up to track the long-term trajectory of the belief shift and to evaluate its sustained impact on self-management and clinical endpoints. Furthermore, the focus on a specialized patient population limits the generalizability of our findings. Subsequent studies should aim to expand the participant pool to different demographic subgroups and other chronic conditions to test the model's broader applicability, utilizing larger sample sizes to robustly assess outcome sustainability. In addition, the multicomponent and culturally specific nature of our intervention, combined with the lack of mediation analysis and objective measures, limits our ability to identify causal mechanisms, core active ingredients, and cross-cultural generalizability. Future research should address these limitations by including mediation analysis, component trials, and objective outcome measures (e.g., medication adherence via electronic monitors). The digital transformation of this intervention via mobile health platforms presents a promising avenue for significantly enhancing its accessibility and scalability in community settings.

## 6 Conclusions

In summary, this study provides evidence that a family empowerment-centered model health education intervention was more effective than standard community education for enhancing blood pressure control and self-management in community-dwelling older adult hypertensive patients with CHLC. Beyond its clinical efficacy, this trial suggests a shift in chronic disease management: from a patient-directed model toward a family-empowered approach. The success of this feasible strategy highlights the critical role of leveraging family support systems within community settings. For policymakers and clinicians, our findings support the consideration of integrating family empowerment principles into standard HTN care protocols for the older adult, offering a practical pathway to improve health outcomes in community health services.

## Data Availability

The original contributions presented in the study are included in the article/supplementary material, further inquiries can be directed to the corresponding author.
